# Impact of Inhaled Nitric Oxide on the Sulfatide Profile of Neonatal Rat Brain Studied by TOF-SIMS Imaging

**DOI:** 10.3390/ijms15045233

**Published:** 2014-03-25

**Authors:** Hanane Kadar, Hoa Pham, David Touboul, Alain Brunelle, Olivier Baud

**Affiliations:** 1 Centre de Recherche de Gif, Institut de Chimie des Substances Naturelles, CNRS, Avenue de la Terrasse, Gif-sur-Yvette Cedex 91198, France; E-Mails: hananekadar@yahoo.fr (H.K.); Alain.Brunelle@cnrs.fr (A.B.); 2 Institut National de la Santé Et de la Recherche Médicale (INSERM) U1141, Université Paris Diderot, PRES Sorbonne Paris-cité, Hôpital Robert Debré, 48 Boulevard Sérurier, Paris 75019, France; E-Mails: hoahp83@yahoo.com (H.P.); olivier.baud@rdb.aphp.fr (O.B.); 3 Réanimation et Pédiatrie Néonatales, Hôpital Robert Debré, 48 Boulevard Sérurier, Paris 75019, France

**Keywords:** myelination, developing brain, white matter, mass spectrometry imaging, TOF-SIMS, sulfatides

## Abstract

Despite advances in neonatal intensive care leading to an increased survival rate in preterm infants, brain lesions and subsequent neurological handicaps following preterm birth remain a critical issue. To prevent brain injury and/or enhance repair, one of the most promising therapies investigated in preclinical models is inhaled nitric oxide (iNO). We have assessed the effect of this therapy on brain lipid content in air- and iNO-exposed rat pups by mass spectrometry imaging using a time-of-flight secondary ion mass spectrometry (TOF-SIMS) method. This technique was used to map the variations in lipid composition of the rat brain and, particularly, of the white matter. Triplicate analysis showed a significant increase of sulfatides (25%–50%) in the white matter on Day 10 of life in iNO-exposed animals from Day 0–7 of life. These robust, repeatable and semi-quantitative data demonstrate a potent effect of iNO at the molecular level.

## 1. Introduction

Dramatic improvements in the perinatal management of preterm neonates have decreased neonatal mortality, but not the incidence of brain damage, leading to cerebral palsy and cognitive/behavioral deficits. Cerebral palsy results from a number of various insults to the developing brain, acting alone or in combination. In recent reports, 5% to 10% of preterm infants developed cerebral palsy, and 25% to 50% had cognitive impairment and behavioral disorders [1–3]. Therefore, the prevention of neurological disabilities remains a major challenge for public health, since no specifically neuroprotective treatment to date has proven clinically useful.

Despite the efforts made to improve knowledge on brain injury and specially to identify predictive biomarkers of later neurodevelopment risks, the understanding of this developmental disease remains limited. Although periventricular white matter (WM) damage is recognized to be one of the main brain lesions leading to neurocognitive impairments, the mechanisms leading to brain injury are not fully elucidated [4]. Hypoxia-ischemia is a common cause of neonatal brain damage, which impacts on cerebral maturation [5]. Inflammation is also a key factor disrupting the developmental program of WM maturation [4]. WM damage is characterized by both focal and diffuse injuries, loss of early differentiating oligodendrocytes and delayed myelination [6,7].

Myelin is an essential component of the WM, representing 40% to 50% of its dry weight. It plays an important role in high-speed conduction, transfer signaling fidelity on long distances and space economy [8]. To fulfill its function, myelin has a unique composition of several specific proteins, such as myelin basic protein (MBP), proteolipid protein (PLP) and a high lipid content (>70%) [9]. Lipids encountered in WM are principally sphingolipids, particularly galactosylceramides and sulfatides. More than 50% of these lipids are hydroxylated at the C-2 carbon of the amide-linked fatty acid [10].

In animal models, conventional biological methods, such as immunohistochemistry, staining and fluorescent tagging, are well established for studying the *in situ* distribution of different species in biological tissues and, particularly, on rat brain slices [5,11]. In the case of myelination default assessment, immunohistochemistry, using various primary antibodies, is usually carried out [12,13]. A limitation of these methods is that they describe the distribution of only one specific compound or cell on a tissue. Contrariwise, mass spectrometry imaging (MSI) gives a simultaneous identification and localization of various organic compounds with a single sample [14–18].

MSI has now emerged as a powerful tool for studying the *in situ* distribution of compounds within various biological tissues. MSI can be obtained from tissue samples by identifying compounds with their mass-to-charge ratio (*m*/*z*). It has already been applied for biomarker discovery [19,20] and biomedical research [21–23]. Nowadays, the three most often implemented techniques to obtain molecular images are, *i.e.*, MALDI (matrix assisted laser desorption ionization), SIMS (secondary ion mass spectrometry) and DESI (desorption electrospray ionization) coupled with various mass analyzers. The typical resolution of DESI is in the range of 100 μm, and for MALDI, the value is generally ~50 μm, reaching, in some case, ~20 μm or less. While the so-called nano-SIMS is dedicated to elemental analyses and reaches spatial resolutions below 100 nm [24], time-of-flight secondary ion mass spectrometry (TOF-SIMS) is more used for molecular analysis of organic samples and can reach a resolution of 500 nm to 2 μm [25,26]. However, for all MSI methods, spatial resolution is a parameter that goes against sensitivity and should be selected wisely. Whereas MALDI needs the sample surface to be homogeneously coated with a matrix, TOF-SIMS imaging requires only the sample to be dried. The development of polyatomic ion sources has greatly improved the capabilities of TOF-SIMS imaging, extending the mass range of analysis by enhancing the efficiency of the intact molecule desorption/ionization, particularly for secondary ions of a mass-to-charge ratio greater than *m*/*z* 300–500 [27,28]. This method has already been successfully applied to lipid analysis in various studies [16,26,29–31].

Inhaled nitric oxide (iNO) is a commonly used therapy in neonatal intensive care units to treat persistent pulmonary hypertension and is one of the most promising molecules for neuroprotection. Nitric oxide (NO) is a small water-soluble molecule that can easily diffuse through tissues. It is recognized to be a mediator in various chronic inflammatory systems, and several studies have shown that the production of NO exerts a dual effect on neuronal survival [32]. This therapy option has been approved in 2001 by the European Medicine Evaluation Agency and the European Commission for neonates with hypoxic respiratory failure. Data on the neurological follow-up of premature infants exposed to iNO remain sparse and unclear. However, growing evidence demonstrates that low doses of iNO not only act locally on the pulmonary vasculature, but also have remote effects on the developing brain under basal or pathological conditions, by modulating WM maturation, inflammation and subsequent brain repair in both developing and adult preclinical models of brain damage [11,33–35]. Both El Gazhi *et al*. and Pansiot *et al*. reported that NO could be neuroprotective in the case of high concentrations of glutamate and excitotoxic damage [34,36]. In the case of developing brain under basal conditions, as previously described by Olivier *et al.* [11], iNO plays a crucial role in the myelination of the developing CNS. Thus, as a preliminary study, TOF-SIMS imaging was implemented to monitor sulfatide level changes after this treatment in normal neonatal brain in rodents. The next step will be to test such an approach in various preclinical models of brain injury.

## 2. Results and Discussion

### 2.1. Imaging Data

**Figure 1.**
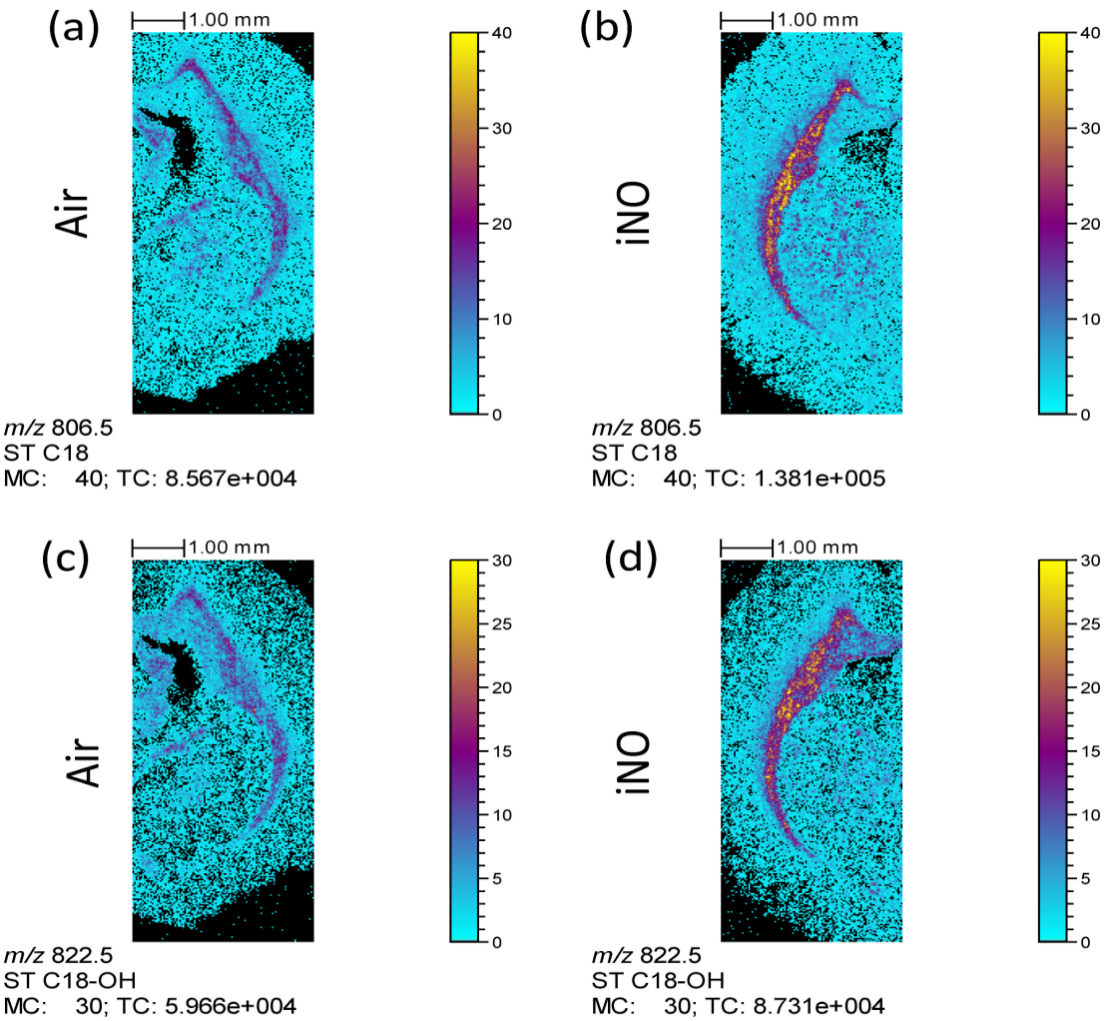
Time-of-flight secondary ion mass spectrometry (TOF-SIMS) negative ion images of coronal rat brain half sections. (**a**) and (**b**): *m*/*z* 806.5 (ST C18) ion; (**c**) and (**d**): *m*/*z* 822.5 (ST C18-OH) ion. These images have been obtained from different rat half brains at postnatal age P10. (**a**) and (**c**): air-exposed rat; (**b**) and (**d**): iNO-exposed rat. Primary ions: Bi_3_^+^, 9.5 × 10^9^ ions cm^−2^; areas varying from 7 mm × 9.5 mm to 8 mm × 11 mm. The maximum number of counts (MC) corresponds to the amplitude of the color scale, and the total counts (TC) are the total number of counts recorded for the specified *m*/*z* (this is the sum of counts in all the pixels).

The ion images (Figure 1) emphasized the variation of myelin composition following rat exposure to iNO previously observed by other techniques, like immunohistochemistry or electron microscopy [11]. Although the sulfatide composition of the WM has previously been determined [37–39], these techniques require the precise dissection of small parts of the brain, prior to the lipid extraction. On the contrary, MSI can offer the opportunity to easily describe the distribution of various endogenous or exogenous compounds within an intact tissue section. TOF-SIMS ion images (Figure 1) of one sulfatide and its hydroxylated form (ST C18 and ST C18-OH) illustrate this statement. Sjövall *et al.* [28] and Pernber *et al.* [40] assigned signals between *m*/*z* 770 and *m*/*z* 910 to glycosphingolipids based on: (1) the comparison with published electrospray ionization mass spectra [41,42] and MSI (MALDI and nanoparticle-assisted laser desorption/ionization) [43,44]; (2) comparison with TOF-SIMS spectra recorded on reference samples containing the pure lipid substances; and (3) calculation of exact masses and isotope distributions of the different substances. This class of lipids represents 25% of the lipid content of myelin [8]. Moreover, Zöller *et al.* [10] demonstrated that sulfatides have a key role in the long-term maintenance of myelin and that the absence of 2-hydroxylated sulfatides can cause myelin sheath degeneration. Figure 1 also highlights the difference in both hydroxylated and non-hydroxylated sulfatide levels in the WM of iNO-treated rats compared to control ones. Indeed, the intensity recorded in the WM is significantly larger in the iNO-exposed rat brain than in the control one. These qualitative results are in agreement with the study of Olivier *et al.* [11], where neonatal exposure to iNO was associated with a transient increase in central nervous system myelination in rats and C57BL/6 mice. Consequently, the iNO treatment can be correlated to the enhancement of the local concentration of hydroxylated sulfatides. These data are consistent with a significant increase in mature oligodendroglial cells density within the developing white matter, as previously reported [11]. These findings are of key importance, shedding light on precious information on the identity of the lipid involved in the treatment to encourage the myelination process.

### 2.2. Semi-Quantitative Approach

**Figure 2.**
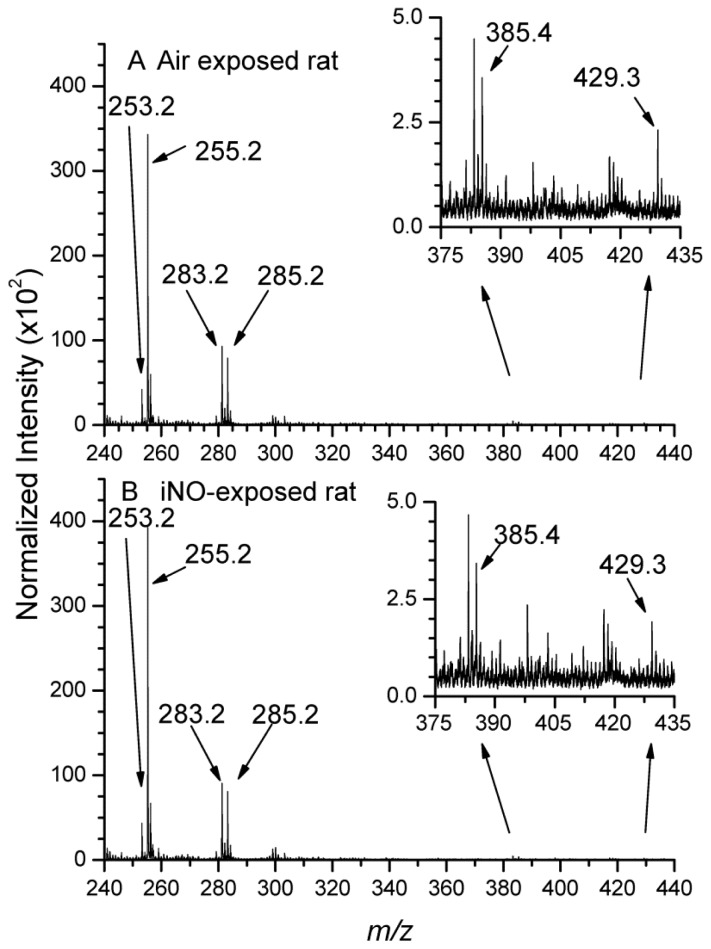
TOF-SIMS mass spectra (*m*/*z* range 240–440) of areas corresponding to the white matter of P10 rat brain tissue sections. (**A**) air-exposed rat; (**B**) iNO-exposed rat.

**Figure 3.**
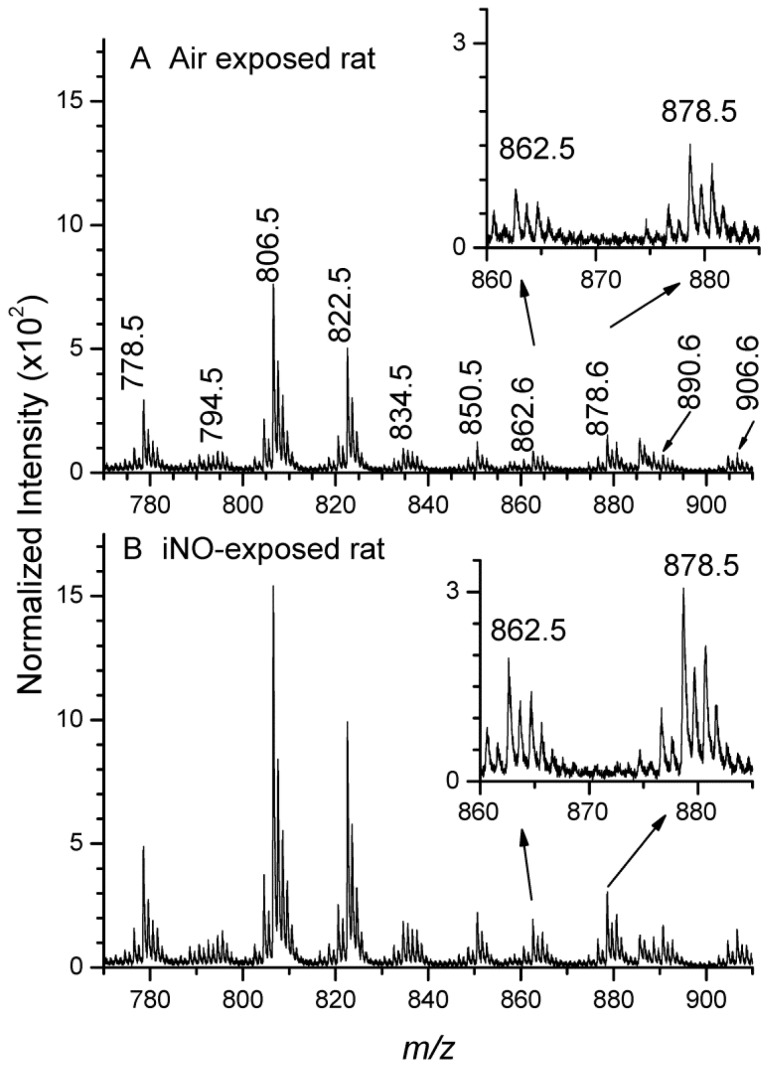
TOF-SIMS mass spectra (*m*/*z* range 770–910) of areas corresponding to the white matter of P10 rat brain tissue sections. (**A**) air-exposed rat; (**B**) iNO-exposed rat.

To better characterize and quantify the variation of the sulfatide level in the WM, triplicate experiments for each group were achieved. The regions of interest (ROIs) were drawn in the polyline mode, by circling the area of the WM, easily distinguishable in the total ion image. Global mass spectra containing all the ions associated with the myelin sheath were then extracted and normalized to equal areas. These spectra were then compared to assess the composition variation between the brains of air-(spectrum A) and iNO-exposed (spectrum B) rat (Figures 2 and 3). The experimental variability of TOF-SIMS mass spectrometry imaging has already been assessed by Bich *et al.* [45], showing that this method could be considered an accurate analytical method for biological studies. Below *m*/*z* 350 (Figure 2), the most inte nse signals have already been assigned in the literature. Indeed, localization of fatty acids in the white matter of the rat brain section has been previously described by TOF-SIMS imaging [28,46,47]). Vitamin E and cholesterol have also been detected in the rat brain section [28,48]. The ion peak intensities of six fatty acids, cholesterol and Vitamin E were then compared for the two groups in order to highlight potential variation (Figure S1). No significant variation was observed, taking into account relative standard deviations. This was confirmed by the Student’s test (*p* > 0.05). We could consequently consider that the composition of fatty acids, cholesterol and Vitamin E is in a steady state in the iNO group compared to the control group.

**Figure 4.**
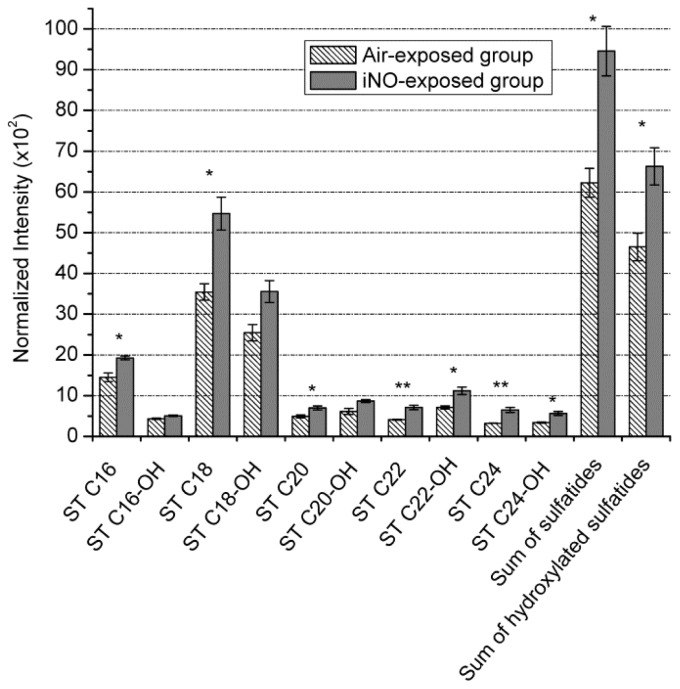
Mean relative intensities of 10 sulfatide ion peaks (non-hydroxylated and hydroxylated) and their sum calculated from a triplicate analysis of P10 rat brain tissues (exposed to air and iNO; *n* = 6 in each experimental group) with their standard deviations. A Student’s test with a confidence interval of 0.05 showed the significant difference between the two groups and for each ion (* for *p*-value ≤ 0.05, ** for *p*-value ≤ 0.01).

We then compared the sulfatide level intensities in the higher mass range (Figure 3). The characteristics of the targeted compounds are displayed in Table 1. After normalization, the intensity of each of the ten sulfatide ion peaks and the sum of both their hydroxylated and non-hydroxylated forms were reported in Figure 4. The Student’s test, applied with a 95% confidence interval, demonstrated the significant intensity increase, ranging from 25% to 50%, for seven different sulfatide species from the air to iNO group; except for ST C16-OH, ST C18-OH and ST C20-OH, showing the same trend, but larger variability. Our results demonstrate that TOF-SIMS, which provides precious information concerning the localization of various species, is also robust and reliable for semi-quantitative biological studies, which require low variability. They confirm that the enhancement of the local concentration of sulfatides in the WM of iNO-treated rats correlated with an increase of the myelination process [10,11].

## 3. Experimental Section

### 3.1. Animals

This study was approved by the National Institute of Health and Medical Research and complied with the instructions of the Institutional Animal Care and Use Committees INSERM 676Paris. One day before delivery, pregnant rats (Sprague-Dawley, Janvier S.A.S, Le Genest-St-Isle, France) were randomized into two experimental groups. The exposed group was placed in a transparent Plexiglas chamber containing 5 parts per million (ppm) of iNO for 7 days (from postnatal day P0 to P7). The air group was kept in a similar Plexiglas chamber without iNO and in the same room. The concentrations of both NO and NO_2_ (lower than 1 ppm) were monitored using a NOXBOX+ system (Bedfont Scientific, Harrietsham, UK). To determine the specific effects of iNO on rat pups, mothers were changed daily from the exposed group to the unexposed experimental group. From P7, rat pups and their mothers in all experimental groups were kept in room air. Animals were housed under controlled temperature (22 ± 1 °C) and light conditions (12 h day/night cycle) with food and water *ad libitum*. Neonatal mortality was checked daily up to sacrifice on P10.

### 3.2. Sample Preparation

After decapitation, brains were harvested and immediately frozen in cold isopentane. The following sample preparation process is routinely used for the MSI of biological tissues in the laboratory [21,48]. In the present case, 14 μm-thick coronal sections from six P10 pups rat brains belonging to two different groups (air- and iNO-exposed animals) were cut at −25 °C using a CM3050-S cryostat (Leica Microsystems SA, Nanterre, France). Coronal sections corresponding to the same interaural depth (2.96 mm) were immediately deposited on silicon wafers (2-in diameter polished silicon wafers, ACM, Villiers-Saint-Frédéric, France). Before analysis, samples were dried under vacuum at a pressure of a few hectopascals for 15 min and were not subjected to any further treatment.

### 3.3. TOF-SIMS Imaging Acquisition

The experiments were performed using a TOF-SIMS IV mass spectrometer (ION-TOF GmbH, Münster, Germany), equipped with a liquid metal ion gun (LMIG) filled with bismuth, allowing it to deliver Bi_n_^q+^ cluster ion beams and, in particular, Bi_3_^+^. Those ions were chosen, since they provide the best compromise between intensity and efficiency combined with a short acquisition time [22]. During the experiments, these primary ions reached the sample surface with an incidence angle of 45°, at a kinetic energy of 25 keV. The pulsed ion current was measured at 10 kHz with a Faraday cup located on the grounded sample holder. Its value was about 0.45 pA, with a pulse duration of less than 1 ns when hitting the sample surface. Then, the produced secondary ions were accelerated to a kinetic energy of 2 keV and flew through a field-free region, where they were reflected with a single stage reflector. Finally, ions were submitted to a post-acceleration of 10 kV just before hitting a hybrid detector made of one single micro-channel plate, followed by a scintillator and a photomultiplier. A low-energy electron flood gun was activated between two primary ions pulses in order to neutralize the sample surface with minimum damage. The effective ion flight path was about 2 m in the time-of-flight tube (reflectron mode), enabling a mass resolution exceeding M/ΔM = 5000 (full width half maximum (FWHM) at *m*/*z* 500).

Due to the very low initial kinetic energy distribution of the secondary ions, the relationship between the time-of-flight and the square root of *m*/*z* is always linear over the whole mass range. Consequently, the mass calibration was initially made with H^−^, C^−^, CH^−^, CH_2_^−^, CH_3_^−^, C_2_^−^, C_3_^−^ and C_4_H^−^ ions in the negative ion mode. To further improve mass accuracy, the mass calibration was refined by adding ions of higher mass, such as fatty acid carboxylates and deprotonated vitamin E [21,22].

Images were acquired in the so-called stage scan mode, with a final spatial resolution of ~8 μm (pixel size). With this mode, the sample was step by step moved by the sample stage in order to scan the whole surface area, patch by patch, each individual patch having an area of 256 μm × 256 μm. Surface areas were similar for each brain hemisphere and set to 3 mm × 8.5 mm. Each image is constituted of ~4.8 × 10^5^ pixels according to the size of the image. The time required to record each image was about 1 h, and the primary ion dose density was fixed to 9.5 × 10^1^ ions/cm^2^, *i.e.*, below the static limit of SIMS, for all the acquisitions.

### 3.4. TOF-SIMS Imaging Processing

Data processing was achieved using Surface Lab 6.2 software (ION-TOF GmbH, Münster, Germany). It allows extractions of ion spectra and images from the raw data. Regions of interest (ROIs) corresponding to specific anatomical brain structures were drawn with the same software. In order to compare the relative intensity of lipids contained in the WM of different samples, the associated mass spectra were further extracted using these ROIs. These selected regions have different areas (in pixels). Therefore, and for a proper comparison, the intensity of the mass spectrum from each ROI was normalized as if it was composed of the same number of pixels as the smallest one. Relative quantifications of compounds showing similar physical and chemical properties (which means desorbed from the sample surface and detected with the same efficiencies) can then be made between different samples, given that all the data have been acquired under the same experimental conditions and that the ROIs have been chosen from equivalent histological areas [26,30,49].

## 4. Conclusions

TOF-SIMS imaging showed its great capabilities for the measurement of the molecular composition of the WM of air- and iNO-exposed rat brains. This method has been proven to produce both qualitative and semi-quantitative results, standing as an attractive complementary method to strengthen the tools available for brain injury research. We have not only identified with precision the presence of sulfatides in the brain WM, but also clearly determined the effect of a promising therapy used in neonates on the myelination process. Other neurological diseases, where alterations of the brain lipid composition can be expected, could also benefit from such an approach. Compared to MALDI, TOF-SIMS is neither the most popular MSI method, nor does it have the highest sensitivity. However, this method is known to produce particularly reproducible results, and moreover, due to its acquisition by a counting system, it is well adapted to semi-quantitative measurements. TOF-SIMS appears to be an attractive complementary method for studying neuroprotection research in diseases where alterations of the brain lipid composition can be expected.

## Supplementary Information



## Figures and Tables

**Table 1.** Assignments of the compounds studied, including complete name, abbreviations, class and sub-class membership, formula, mass spectrometric diagnostic species, calculated and measured values of *m*/*z* and error in parts per million of the detected [M-H]^−^ ion species.

Species	Abbreviations	Formula	Calculated *m*/*z*	Experimental *m*/*z*	Error (ppm)
(3′-Sulf)Galβ-Cer(d18:1/16:0)	ST C16	C_40_H_76_SNO_11_	778.51	778.52	13
(3′-Sulf)Galβ-Cer(d18:1/2-OH-16:0)	ST C16-OH	C_40_H_76_SNO_12_	794.51	794.53	25
(3′-Sulf)Galβ-Cer(d18:1/18:0)	ST C18	C_42_H_80_SNO_11_	806.54	806.54	0
(3′-Sulf)Galβ-Cer(d18:1/2-OH-18:0)	ST C18-OH	C_42_H_80_SNO_12_	822.54	822.55	12
(3′-Sulf)Galβ-Cer(d18:1/20:0)	ST C20	C_44_H_84_SNO_11_	834.57	834.55	−24
(3′-Sulf)Galβ-Cer(d18:1/2-OH-20:0)	ST C20-OH	C_44_H_84_SNO_12_	850.57	850.56	−12
(3′-Sulf)Galβ-Cer(d18:1/22:0)	ST C22	C_46_H_88_SNO_11_	862.61	862.60	−12
(3′-Sulf)Galβ-Cer(d18:1/2-OH-22:0)	ST C22-OH	C_46_H_88_SNO_12_	878.60	878.59	−11
(3′-Sulf)Galβ-Cer(d18:1/24:0)	ST C24	C_48_H_92_SNO_11_	890.64	890.62	−22
(3′-Sulf)Galβ-Cer(d18:1/2-OH-24:0)	ST C24-OH	C_46_H_92_SNO_12_	906.63	906.61	−22

## References

[1] Larroque B., Ancel P.Y., Marchand-Martin L., Cambonie G., Fresson J., Pierrat V., Roze J.C., Marpeau L., Thiriez G., Alberge C. (2010). Special care and school difficulties in 8-year-old very preterm children: The epipage cohort study. PLoS One.

[2] Woodward L.J., Clark C.A.C., Bora S., Inder T.E. (2012). Neonatal white matter abnormalities an important predictor of neurocognitive outcome for very preterm children. PLoS One.

[3] Woodward L.J., Moor S., Hood K.M., Champion P.R., Foster-Cohen S., Inder T.E., Austin N.C. (2009). Very preterm children show impairments across multiple neurodevelopmental domains by age 4 years. Arch. Dis. Child-Fetal Neonatal Ed.

[4] Volpe J.J. (2009). Brain injury in premature infants: A complex amalgam of destructive and developmental disturbances. Lancet Neurol.

[5] Zhang Y.P., Huang Q.L., Zhao C.M., Tang J.L., Wang Y.L. (2011). GM1 improves neurofascin 155 association with lipid rafts and prevents rat brain myelin injury after hypoxia-ischemia. Braz. J. Med. Biol. Res.

[6] Inder T., Neil J., Kroenke C., Dieni S., Yoder B., Rees S. (2005). Investigation of cerebral development and injury in the prematurely born primate by magnetic resonance imaging and histopathology. Dev. Neurosci.

[7] Khwaja O., Volpe J.J. (2008). Pathogenesis of cerebral white matter injury of prematurity. Arch. Dis. Child-Fetal.

[8] Baumann N., Pham-Dinh D. (2001). Biology of oligodendrocyte and myelin in the mammalian central nervous system. Physiol. Rev.

[9] Siegel J.S., Wayne Albers R., Brady S., Price D. (2006). Basic Neurochemistry, Molecular, Cellular and Medical Aspects.

[10] Zöller I., Meixner M., Hartmann D., Bussow H., Meyer R., Gieselmann V., Eckhardt M. (2008). Absence of 2-hydroxylated sphingolipids is compatible with normal neural development but causes late-onset axon and myelin sheath degeneration. J. Neurosci.

[11] Olivier P., Loron G., Fontaine R.H., Pansiot J., Dalous J., Thi H.P., Charriaut-Marlangue C., Thomas J.L., Mercier J.C., Gressens P. (2010). Nitric Oxide Plays a Key Role in Myelination in the Developing Brain. J. Neuropathol. Exp. Neurol.

[12] Olivier P., Baud O., Bouslama M., Evrard P., Gressens P., Verney C. (2007). Moderate growth restriction: Deleterious and protective effects on white matter damage. Neurobiol. Dis.

[13] Dean J.M., Farrag D., Zahkouk S.A.M., El Zawahry E.Y.I., Hagberg H., Kjellmer I., Mallard C. (2009). Cerebellar white matter injury following systemic endotoxemia in preterm fetal sheep. Neuroscience.

[14] Setou M. (2010). Imaging Mass Spectrometry.

[15] Rubakhin S.S., Sweedler J.V. (2010). Mass Spectrometry Imaging, Principles and Protocols.

[16] Wu C., Dill A.L., Eberlin L.S., Cooks R.G., Ifa D.R. (2013). Mass spectrometry imaging under ambient conditions. Mass Spectrom. Rev.

[17] Gode D., Volmer D.A. (2013). Lipid imaging by mass spectrometry. Analyst.

[18] Brunelle A., Touboul D., Laprévote O. (2007). Recent advances in biological tissue Imaging with time-of-flight secondary ion mass spectrometry: Polyatomic ion sources, sample preparation, and applications. Curr. Pharm. Des.

[19] Cazares L.H., Troyer D.A., Wang B., Drake R.R., Semmes O.J. (2011). MALDI tissue imaging: From biomarker discovery to clinical applications. Anal. Bioanal. Chem.

[20] Bakry R., Rainer M., Huck C.W., Bonn G.K. (2011). Protein profiling for cancer biomarker discovery using matrix-assisted laser desorption/ionization time-of-flight mass spectrometry and infrared imaging: A review. Anal. Chim. Acta.

[21] Touboul D., Brunelle A., Halgand F., de La Porte S., Laprévote O. (2005). Lipid imaging by gold cluster time-of-flight secondary ion mass spectrometry: Application to Duchenne muscular dystrophy. J. Lipid Res.

[22] Tahallah N., Brunelle A., de La Porte S., Laprévote O. (2008). Lipid mapping in human dystrophic muscle by cluster-time-of-flight secondary ion mass spectrometry imaging. J. Lipid Res.

[23] Gemoll T., Roblick U.J., Habermann J.K. (2011). MALDI mass spectrometry imaging in oncology. Mol. Med. Rep.

[24] Guerquin-Kern J.L., Wu T.D., Quintana C., Croisy A. (2005). Progress in analytical imaging of the cell by dynamic secondary ion mass spectrometry (SIMS microscopy). Biochim. Biophys. Acta.

[25] Passarelli M.K., Winograd N. (2011). Lipid imaging with time-of-flight secondary ion mass spectrometry (ToF-SIMS). Biochim. Biophys. Acta.

[26] Touboul D., Laprévote O., Brunelle A. (2011). Micrometric molecular histology of lipids by mass spectrometry imaging. Curr. Opin. Chem. Biol.

[27] Touboul D., Halgand F., Brunelle A., Kersting R., Tallarek E., Hagenhoff B., Laprévote O. (2004). Tissue molecular ion imaging by gold cluster ion bombardment. Anal. Chem.

[28] Sjövall P., Lausmaa J., Johansson B. (2004). Mass spectrometric imaging of lipids in brain tissue. Anal. Chem.

[29] Touboul D., Brunelle A., Germain D.P., Laprévote O. (2007). A new imaging technique as a diagnostic tool: Mass spectrometry. Presse Med.

[30] Debois D., Bralet M.P., le Naour F., Brunelle A., Laprévote O. (2009). *In situ* lipidomic analysis of nonalcoholic fatty liver by cluster TOF-SIMS imaging. Anal. Chem.

[31] Seyer A., Cantiello M., Bertrand-Michel J., Roques V., Nauze M., Bezirard V., Collet X., Touboul D., Brunelle A., Comera C. (2013). Lipidomic and spatio-temporal imaging of fat by mass spectrometry in mice duodenum during lipid digestion. PLoS One.

[32] Calabrese V., Cornelius C., Rizzarelli E., Owen J.B., Dinkova-Kostova A.T., Butterfield D.A. (2009). Nitric oxide in cell survival: A janus molecule. Antioxid Redox Sign.

[33] Charriaut-Marlangue C., Bonnin P., Gharib A., Leger P.L., Villapol S., Pocard M., Gressens P., Renolleau S., Baud O. (2012). Inhaled nitric oxide reduces brain damage by collateral recruitment in a neonatal stroke model. Stroke.

[34] Pansiot J., Loron G., Olivier P., Fontaine R., Charriaut-Marlangue C., Mercier J.C., Gressens P., Baud O. (2010). Neuroprotective effect of inhaled nitric oxide on excitotoxic-induced brain damage in neonatal rat. PLoS One.

[35] Terpolilli N.A., Kim S.W., Thal S.C., Kataoka H., Zeisig V., Nitzsche B., Klaesner B., Zhu C.L., Schwarzmaier S., Meissner L. (2012). Inhalation of nitric oxide prevents ischemic brain damage in experimental stroke by selective dilatation of collateral arterioles. Circ. Res.

[36] El Ghazi F., Desfeux A., Brasse-Lagnel C., Roux C., Lesueur C., Mazur D., Remy-Jouet I., Richard V., Jégou S., Laudenbach V. (2012). NO-dependent protective effect of VEGF against excitotoxicity on layer VI of the developing cerebral cortex. Neurobiol. Dis.

[37] O’Brien J.S., Sampson E.L. (1965). Fatty acid and fatty aldehyde composition of the major brain lipids in normal human gray matter, white matter, and myelin. J. Lipid Res.

[38] Ramsey R.B., Scott T., Banik N.L. (1977). Fatty acid composition of myelin isolated from the brain of a patient with cellular deficiency of co-enzyme forms of vitamin B12. J. Neurol. Sci.

[39] Marbois B.N., Faull K.F., Fluharty A.L., Raval-Fernandes S., Rome L.H. (2010). Analysis of sulfatide from rat cerebellum and multiple sclerosis white matter by negative ion electrospray mass spectrometry. Biochim. Biophys. Acta.

[40] Pernber Z., Richter K., Mansson J.E., Nygren H. (2007). Sulfatide with different fatty acids has unique distributions in cerebellum as imaged by Time-Of-Flight Secondary Ion Mass Spectrometry (TOF-SIMS). Biochim. Biophys. Acta.

[41] Han X., Holtzman D.M., McKeel D.W., Kelley J., Morris J.C. (2002). Substantial sulfatide deficiency and ceramide elevation in very early Alzheimer’s disease: Potential role in disease pathogenesis. J. Neurochem.

[42] Taguchi R., Hayakawa J., Takeuchi Y., Ishida M. (2000). Two-dimensional analysis of phospholipids by capillary liquid chromatography/electrospray ionization mass spectrometry. J. Mass Spectrom.

[43] Ageta H., Asai S., Sugiura Y., Goto-Inoue N., Zaima N., Setou M. (2009). Layer-specific sulfatide localization in rat hippocampus middle molecular layer is revealed by nanoparticle-assisted laser desorption/ionization imaging mass spectrometry. Med. Mol. Morphol.

[44] Yuki D., Sugiura Y., Zaima N., Akatsu H., Hashizume Y., Yamamoto T., Fujiwara M., Sugiyama K., Setou M. (2011). Hydroxylated and non-hydroxylated sulfatide are distinctly distributed in the human cerebral cortex. Neuroscience.

[45] Bich C., Touboul D., Brunelle A. (2013). Study of experimental variability in TOF-SIMS mass spectrometry imaging of biological samples. Int. J. Mass Spectrom.

[46] Börner K., Malmberg P., Mansson J.E., Nygren H. (2007). Molecular imaging of lipids in cells and tissues. Int. J. Mass Spectrom.

[47] Richter K., Nygren H., Malmberg P., Hagenhoff B. (2007). Localization of fatty acids with selective chain length by imaging time-of-flight secondary ion mass spectrometry. Microsc. Res. Tech.

[48] Benabdellah F., Seyer A., Quinton L., Touboul D., Brunelle A., Laprévote O. (2010). Mass spectrometry imaging of rat brain sections: Nanomolar sensitivity with MALDI versus nanometer resolution by TOF-SIMS. Anal. Bioanal. Chem.

[49] Malmberg P., Jennische E., Nilsson D., Nygren H. (2011). High-resolution, imaging TOF-SIMS: Novel applications in medical research. Anal. Bioanal. Chem.

