# Supplementary material for: Impact of Inhaled Nitric Oxide on the Sulfatide Profile of Neonatal Rat Brain Studied by TOF-SIMS Imaging
Source: Int J Mol Sci. 2014 Mar 25;15(4):5233–45. doi: 10.3390/ijms15045233 (PMC4013560; doi:10.3390/ijms15045233)
Supplement: Supplementary file 1 [file ijms-15-05233-s001.pdf]

Supplementary Information

Figure S1. Mean relative ion peak intensities of 6 fatty acids, cholesterol and Vitamin E calculated from a triplicate analysis of P10 rat brain tissues (exposed to air and iNO) with their standard deviations. No significant variation was observed as analyzed using a student test ($p > 0.05$).

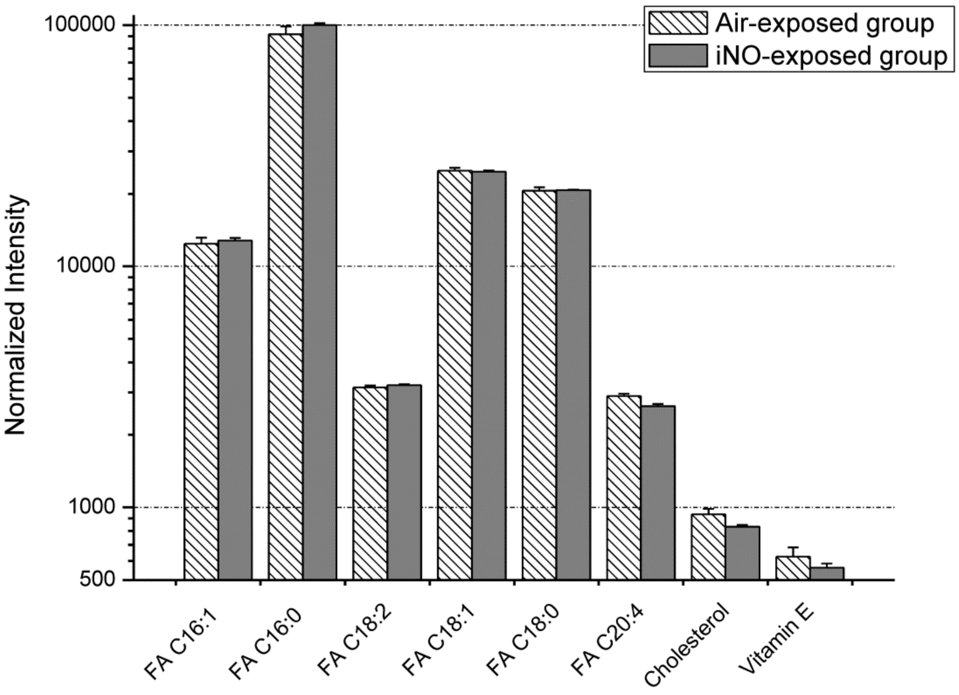

© 2014 by the authors; licensee MDPI, Basel, Switzerland. This article is an open access article distributed under the terms and conditions of the Creative Commons Attribution license (http://creativecommons.org/licenses/by/3.0/).
